# The Intimate and Sexual Costs of Emotional Labor: The Development of the Women’s Sexual Emotional Labor Assessment

**DOI:** 10.1007/s10508-024-03061-7

**Published:** 2024-12-19

**Authors:** Tanja Oschatz, Jennifer L. Piemonte, Verena Klein

**Affiliations:** 1 Institute for Social and Legal Psychology, Johannes Gutenberg University, Binger Strasse 14-16, 55122 Mainz, Germany; 2 Department of Psychology, University of Illinois, Urbana-Champaign, IL USA; 3 School of Psychology, University of Southampton, Southampton, UK

**Keywords:** Emotional labor, Sexual pleasure, Gender inequality, Women’s sexuality, Close relationships

## Abstract

Emotional labor, the act of suppressing or altering one’s feelings to enhance another person’s well-being, is predominantly performed by women, especially within intimate relationships. Despite its impact on well-being, research on its role in sexuality remains limited. We developed the Women’s Sexual Emotional Labor Assessment (WOSELA), a 12-item scale based on themes identified by past qualitative studies. To ensure its reliability and validity, we conducted three studies with women partnered with men, as well as heterosexual single women from the UK and the USA (*N* = 831). Drawing on exploratory factor analysis in Study 1, four subscales were identified: faking orgasm, performing desire, tolerating discomfort or pain, and partner-referenced sexual satisfaction. Confirmatory factor analyses in Study 2 (pre-registered) provided support for the WOSELA in its current form. In Study 3 (pre-registered), we demonstrated the validity of the WOSELA by establishing significant negative correlations with sexual agency, sexual communication, and sexual pleasure. Our results further show that the WOSELA was not significantly related to positive-partner-oriented sexual responsiveness. The WOSELA offers a unique tool to explore the role of emotional labor in sustaining the pleasure gap, as well as in broader power dynamics within intimate relationships and its impact on women’s well-being.

**Supplementary Information:**

The online version contains supplementary material available at 10.1007/s10508-024-03061-7.

## Introduction

In many Western countries, gender egalitarianism in long-term relationships has become a cultural ideal, with couples aiming for equitable power distributions within their partnerships (Bay-Cheng et al., 2018; Carlson et al., 2016; Gerson, 2010; Rostosky & Riggle, 2017). This ideal also transfers to the realm of sexuality, where both women and men show a preference for sexual relationships in which the partners experience relatively equal sexual pleasure, a finding that has been apparent for the past three decades (Döring & Mohseni, 2022; Rubin, 1990). However, despite these aspirations, a substantial disparity in sexual pleasure experiences between women and men persists, especially in heterosexual relationships (Conley & Klein, 2022; Laan et al., 2021). Research consistently shows that, in heterosexual contexts, women tend to experience fewer orgasms (Frederick et al., 2018; Garcia et al., 2014; Piemonte et al., 2019), less overall pleasure (Herbenick et al., 2010), and more painful or negative encounters compared to men (Elmerstig et al., 2013; Gordon & Riger, 2011).

These findings emphasize the significance of recognizing that gender inequality in a couple’s sexual relationship—similar to other aspects of their lives such as housework or childcare—continues to be an issue that disproportionately disadvantages women (Craig & Churchill, 2021; Daminger, 2020). We draw on psychological and sociological theories and posit that, just like in other realms of relational life, the concept of emotional labor constitutes a fundamental, yet often hidden, component in the perpetuation of gender inequality in sexuality (Dean et al., 2022; Fahs & Swank, 2016). Given the limited research attention to the phenomenon of sexual emotional labor, the goal of the present research is to introduce a scale that can effectively measure this construct, thus providing a valuable tool for future research.

### Emotional Labor and Its Gendered Nature

Emotional labor is a multifaceted construct that involves engaging in actions aimed at enhancing the emotional well-being of others and providing them with emotional support (Erickson, 1993). These actions often require one to either “induce or suppress feelings in order to sustain outward countenance” (Hochschild, 1983, p. 7). Therefore, emotional labor has a performative element that drives people to bring their emotions in alignment with another person, but also with cultural expectations of what they think they are expected to feel (Elliott & Umberson, 2008; Hochschild, 1983). The concept includes both so-called “surface acting,” meaning the display of emotions without genuinely feeling them, and “deep acting,” where one tries to convince themselves that they are actually feeling the emotion expected of them (Hochschild, 1983). Emotional labor is not always a conscious process, but rather standardized, automatic, and often understood as a mandatory element to sustain relationships (Dean et al., 2022; Fahs & Swank, 2016).

The concept was introduced by Hochschild (1983) in a study of the emotional demands and expectations placed on workers in US service industries. Since then, it has been extended and applied to various aspects of life beyond the workplace, including intimate relationships or family dynamics. Hochschild argued that due to gendered expectations, women may be more vulnerable to suppressing their feelings to enhance the well-being of others as compared to men. Indeed, numerous studies have indicated that women tend to assume a disproportionate amount of emotional labor in areas like running the household (Zimmerman et al., 2002), and mothers were observed exerting intense emotional labor during the COVID-19 pandemic to maintain a sense of safety and calmness for their family members (Hjálmsdóttir & Bjarnadóttir, 2021). This pattern also occurs in the workplace (Guy & Newman, 2004), where female professors have been found to employ intense emotional labor by behaving more friendly, caring and encouraging toward their students compared to their male counterparts (Bellas, 1999).

### Social Role Theory and Emotional Labor

One theory that explains these gender differences in the performance of emotional labor is social role theory (Eagly, 1987). This perspective ascribes gender differences to the labor divisions in society that indirectly form gendered social roles, such as women’s reproductive labor or men’s military labor (Grijalva et al., 2015). Although these roles have looked differently throughout history, patriarchal structures persist in which men have more access to social, economic, and political power than women, who disproportionately occupy subordinate workplace and household positions (Eagly & Wood, 1999, 2012).

Psychologically speaking, characteristics associated with these distinct social roles have become intertwined with the genders associated with their respective roles. That is, the gendered division of labor in Western society has given rise to gender stereotypes that include agentic traits for men and communal traits, including the propensity to engage in emotional labor, for women (Grijalva et al., 2015). As a result, actions involving emotional labor are frequently seen as instinctive and natural manifestations of women’s affection and care (MacDonald et al., 1993). Deviating from gender norms can lead to the so-called backlash effect, implying social or economic sanctioning, particularly for women (Rudman, 1998). Complementing this theory, the “doing gender” approach suggests that individuals conform to gender normative behaviors not only to avoid potential backlash, but also because they are motivated to enact symbolic femininity or masculinity. This can involve either avoiding or engaging in behaviors associated with female or male traits to align with societal expectations of gender roles (West & Zimmerman, 1987; Zamberlan et al., 2021).

### Emotional Labor in Intimate Relationships

Fewer studies have looked at emotional labor in the contexts of romantic relationships. In an early sociological analysis, Duncombe and Marsden (1993) underscored the relevance of researching the phenomenon as a factor alongside other gendered instrumental aspects, like gender inequalities in financial management or domestic labor. Suggesting that various power distributions within a relationship may be interconnected, researchers found a positive correlation between the amount of housework carried out and the amount of emotional labor performed within the relationship, suggesting that if a person performs the majority of the housework, they are also likely to perform the majority of emotional labor (Minnotte et al., 2007). Emotional labor within partnerships manifests in various ways, like caring for a partner when they are unwell, comforting them when they are stressed, or supporting them when they have a problem (Duncombe & Marsden 1993; Erickson, 1993). Like in other spheres, extensive research consistently demonstrates that, in heterosexual contexts, women tend to perform a greater share of emotional labor in their romantic relationships compared to their partners (Duncombe & Marsden, 1993; Umberson et al., 2015), with one study showing that this is especially true if they embraced a feminine gender identity or adhere to traditional feminine roles (Erickson, 2005). A study centered on heterosexual couples with young children found a significant inequality such that fewer than 6% of men reported being more engaged in emotional labor than their partners, whereas a majority of women, over 50%, shouldered the greater responsibility (Strazdins & Broom, 2004).

Gender inequalities in emotional labor might have a negative effect on women’s well-being. Although some studies have found that emotional labor is positively related to relationship satisfaction (Curran et al., 2015; Horne & Johnson, 2019), other studies suggest that persistently directing efforts toward enhancing a partner’s well-being leads to psychological distress, decreased levels of love, and heightened relationship conflict, particularly among women (Strazdins & Broom, 2004; Umberson et al., 2015). Indeed, an unequal distribution of emotional labor appears particularly draining. In a study that examined the allocation of emotional labor in conjunction with other unequal labor distributions (e.g., housework and childcare), only the unequal division of emotional labor was significantly associated with higher levels of distress (Strazdins & Broom, 2004).

### Sexual Emotional Labor

The theoretical considerations of social role theory set out above can aid in understanding gender differences in emotional labor within sexuality. Generally, feminine gender norms are associated with characteristics like friendliness, deference, and caring for others (Eagly & Wood, 2012; Hochschild, 1983). In the context of sexuality, this translates to the traditional female sexual script, which includes notions of passivity, submissiveness, not wanting to be rude or demanding, prioritizing being desirable rather than having desire, and aiming to please the sexual partner (Bowleg et al., 2015; Carter et al., 2019; Klein et al., 2019; Muehlenhard & Shippee, 2010; Rubin et al., 2019; Willis et al., 2018). Therefore, women have often been assigned the role of “pleasure givers,” where their own sexual satisfaction takes a backseat to fulfilling their partner’s desires (Elliott & Umberson, 2008; Fahs & Swank, 2011; Muehlenhard & Shippee, 2010). By adhering to these gender norms and expectations, women may experience certain advantages including social acceptance and approval, increased relationship functioning, and decreased cognitive dissonance (Klein & Conley, 2022; Sanchez et al., 2005). However, these roles and norms also shape expectations regarding women’s sexual behavior such that women are saddled with the job of caretaking both partners’ emotions during partnered sexual experiences. Importantly, the gendered norms leading to emotional labor can be deeply ingrained to the point that individuals are often unaware of engaging in this behavior, rendering the performance of emotional labor largely invisible (Hochschild, 1983; West & Zimmerman, 1987).

Despite the invisibility, pretending to have an emotion or trying to generate a certain emotion that one does not actually feel, for the benefit of someone else, is highly present in many women’s sexualities (Fahs & Swank, 2016). Terms like “sex work” (Duncombe & Marsden, 1993) or “relational sex work” (Cacchioni, 2007) were employed in the research literature early on to describe women’s efforts in managing their partner’s sexual desires and affective responses by adjusting their own emotions. This form of emotional labor can manifest in various ways, such as faking orgasms (Fahs & Swank, 2016; Garcia et al., 2014), or engaging in an unwanted sexual encounter (Impett & Peplau, 2003). Other aspects may include refraining from labeling coercion as rape or accepting a lack of orgasm reciprocity (Fahs & Swank, 2011; Klein & Conley, 2022). Many of those behaviors occur because women want their partners to feel confident and loved, and/or they are fearful of expressing their actual sexual needs (Fahs & Swank, 2011; Faulkner & Lannutti, 2010). In an US community sample of 20 women representing various sexual orientations, ethnicities, and age groups, results revealed that almost all participants had engaged in some form of emotional labor in their sexual experiences (Fahs & Swank, 2016). Notably, emotional labor appears to be more prevalent for women with male sexual partners compared to women with female sexual partners (Fahs & Swank, 2016). This observation underscores the influence of gendered processes within heterosexual relationships, and specifically within mixed-gender sexual encounters, as the crucial context of this phenomenon.

### Themes of Sexual Emotional Labor

To effectively evaluate and assess the performance of sexual emotional labor in women, the availability of a reliable measurement tool is essential. For our scale development, we defined sexual emotional labor as behaviors that are enacted to promote the well-being of a partner, while neglecting one’s own feelings or pleasure experience. Given research indicating that women are particularly likely to perform this type of labor, and the fact that certain emotional labor behaviors in sexuality may be specific to women (Elliott & Umberson, 2008; Fahs & Swank, 2016), our focus of the scale development was directed toward the social group *women*, specifically this research was limited to cisgender women.

Through an in-depth review of several qualitative studies, we identified five major themes of sexual emotional labor that were frequently included in the papers’ analyses and deemed applicable to a majority of women: faking orgasm, tolerating discomfort or pain, accepting dissatisfying or bad sex, defining sexual satisfaction based on partner’s pleasure, and performing desire (Elliott & Umberson, 2008; Fahs & Swank, 2016; McClelland, 2010, 2014). These themes served as guiding categories for item development of our scale. In the following sections, we will provide a brief introduction to each of these themes.

#### Faking Orgasm

Faking orgasms has been subject to numerous studies and may compose one of the most obvious manifestations of sexual emotional labor. Research has consistently found that women are far more likely to fake orgasms as compared to men (Garcia et al., 2014), with prevalence rates of women faking orgasms exceeding 50% across many studies (Darling & Davidson, 1986; Muehlenhard & Shippee, 2010; Opperman et al., 2014). Some women even engage in consistent faking of orgasms (Fahs & Swank, 2011) and this behavior is observed across different cultural contexts (Ford et al., 2022). It is worth noting that not all instances of faking orgasm can be classified as emotional labor, as reasons for faking may also be self-focused (e.g., to increase own arousal; Barnett et al., 2019; Cooper et al., 2014). However, partner-focused or situational reasons, like reassuring one’s partner of his sexual competence and preventing him from feeling inadequate, have been identified as the most common motivations for faking orgasms in hetero sex (Barnett et al., 2019; Nicolson & Burr, 2003). Adding to this understanding, research indicates that women’s orgasms play a pivotal role in affirming men’s sense of masculinity (Chadwick & van Anders, 2017). Consistent with this, women who fake orgasms have a higher desire to please their partner and more difficulties communicating their own needs (Wiederman, 1997), feel more pressure to ensure partner’s satisfaction (Fahs & Swank, 2011), and are more likely to believe that female orgasms are necessary for men’s sexual satisfaction (Harris et al., 2019).

#### Tolerating Pain or Discomfort

Fahs and Swank (2016) identified the practice of tolerating or even suppressing feelings of pain or physical discomfort during sex to avoid making their partner uncomfortable. It is common for girls and women to perceive a connection between sex and pain, often rooted in societal messages and cultural beliefs (Conley & Klein, 2022). From a young age on, girls and young women often learn that sex, and especially “losing” their virginity, can be a painful experience that may (or even should) cause discomfort or bleeding (Carpenter, 2001; Thompson, 1990). Recent studies have shed light on the prevalence of pain during sexual intercourse among women partnered with men. In a nationally representative U.S. sample, almost 30% of women reported painful sexual experiences during penile-vaginal intercourse, compared to only 7% of men (Herbenick et al., 2015). Furthermore, 25% of women described their most recent sexual encounter as physically painful (Carter et al., 2019). Data from Sweden indicate even higher numbers, with nearly half of women reporting painful experiences during intercourse (Elmerstig et al., 2013). Importantly, research shows that women do not only experience discomfort or pain during intercourse. Qualitative studies describe women’s experiences of aversion to the smell or taste of men’s genitals when performing fellatio (Lewis & Marston, 2016) and pain while receiving cunnilingus (Sovetkina et al., 2017). Over half of women do not mention this pain to their partners and a majority report continuing to have sex despite being in pain (Carter et al., 2019; Elmerstig et al., 2013). The most common reasons for not discussing pain or discomfort with their partners include prioritizing their partner’s pleasure, not wanting to disrupt the sexual activity, or fearing that it may hurt their partner’s feelings (Elmerstig et al., 2013). Other explanations revolved around normalizing pain or avoiding uncomfortable or “awkward” situations (Carter et al., 2019). That their concerns revolve around their partners’ pleasure, satisfaction, and emotions highlights the affective and cognitive loads that women take on in prioritizing their partners’ experience over their own.

#### Accepting Dissatisfying or Bad Sex

Another form of emotional labor in sexuality is women’s inclination to expect, accept, and tolerate what is referred to as “bad sex” (Farvid & Braun, 2017). “Bad sex” can be understood as sexual encounters lacking pleasure or involving negative emotions such as anxiety, boredom, or sadness (Fahs & Swank, 2016). In an interview study, some women even described it as feeling alienated from themselves and their partners during the sexual act (Fahs & Swank, 2016). Importantly, this theme should not be misunderstood as passive acceptance. Fahs and Swank emphasized the performative, effortful element at the core of pretending to enjoy “bad sex” while suppressing one’s negative emotions. Remarkably, nearly all interviewed women in their study had encountered unsatisfying sexual experiences characterized by a lack of orgasm, yet they endured it to fulfill what they perceived as their partners’ desires for feelings of power, masculinity, or sexual proficiency. In another study, 59% of women expressed fear that their partners would be disappointed if they did not appear to enjoy penile-vaginal intercourse, leading them to feign sexual pleasure (Elmerstig et al., 2013). These findings highlight the prevalence of women accepting “bad sex” to cater to their partners’ expectations at the expense of their own, genuine thoughts and feelings. Interestingly, different forms of emotional labor may reinforce one another, as one study found that women experiencing less pleasure during sex were also less likely to tell their partner about experiencing sexual pain (Carter et al., 2019).

#### Defining Sexual Satisfaction Based on Partner’s Pleasure

Extensive research suggests that women are likely to focus on their partners’ satisfaction when assessing their own sexual satisfaction. In other words, when women are asked how sexually satisfied they are, they often consider their partner’s sexual satisfaction when reporting their own (McClelland, 2010, 2014). This tendency was also demonstrated in a study where approximately one-third of the interviewed women mentioned that their sexual satisfaction relied heavily on their partner’s pleasure rather than their own (Fahs & Swank, 2016). Similarly, in focus group discussions, women identified *autonomy* as a central theme in defining solitary sexual pleasure, whereas partnered pleasure was predominantly characterized by the theme of *giving pleasure* (Goldey et al., 2016). This practice of placing one’s own desires and wishes secondary to those of a sexual partner is much more commonly found among women than men (Freihart et al., 2021).

From the perspective of social role theory, it may be that women include their partner’s pleasure in their definition of their own sexual satisfaction because they have internalized the communal characteristics associated with the feminine gender role. For instance, women who hold traditional gender beliefs are less likely to assert their own sexual pleasure and consequently experience fewer orgasms (Harris et al., 2016). Furthermore, women who are exposed to benevolent sexism, which describes seemingly positive gender beliefs about women that, in reality, undermine their power, competence, and autonomy, tend to be more likely to engage in sex for relational purposes rather than personal pleasure (Fitz & Zucker, 2015). This partner orientation, which often entails self-neglecting elements, may escapsulate the essence of emotional labor.

#### Performing Desire

Lastly, the act of performing desire has been shown to be a highly prevalent theme of emotional labor in sexuality (Vannier & O’Sullivan, 2010). Performing desire involves expressing interest in, and engaging in, partnered sexuality or specific sexual behaviors despite lacking the genuine wish to do so, and is a more commonly observed phenomenon among women than men (Bay-Cheng & Eliseo-Arras, 2008; Braksmajer, 2017; Impett & Peplau, 2003). In one study, 65% of women as compared to 40% of men stated that they had agreed on having sex although they did not want to at least once in their lives (Impett & Peplau, 2003). Another study of college students found that 16% of women but only 2% of men reported complying with unwanted sex within the first two months on campus (Katz et al., 2012). The act of performing desire occurs across both casual encounters and committed relationships. For example, a study focusing on college students found that 33% of women had complied to unwanted casual sex (Katz & Schneider, 2015). In comparison, a study of women in committed romantic relationships revealed that 65% had experienced unwanted sex with their most recent partner at least once (Himanen & Gunst, 2024).

Research shows that the most common motives of engaging in unwanted sex are to satisfy the needs of the partner, promote intimacy, avoid relationship tension (O’Sullivan & Allgeier, 1998), and help the partner feel better about him or herself (Elliott & Umberson, 2008). Furthermore, many women described the performance of desire as an obligation to maintain their relationship (Elliott & Umberson, 2008). In an interview study of married couples, the majority of women stated that they had made efforts in the past to alter or manipulate their feelings in order to experience more desire for sex with their partners (Elliott & Umberson, 2008). Women were not mistaken in doing so: most men expected their female partners to express desire and show more interest in sex (Elliott & Umberson, 2008). Moreover, the study showed that women believed their male partners had a “need” for sex, which led them to exaggerate their own desire and agree to unwanted sex, a finding corroborated in additional research (Carter et al., 2019; Séguin & Blais, 2021).

This theme also refers to engaging in specific sexual behaviors despite a lack of genuine desire for them. In a community sample of 20 women, one quarter of them described complying to anal or oral sex because they perceived that compliance was expected, although they did not enjoy it (Fahs & Swank, 2021). Further, in a study on casual sex, the results showed that women were more likely than men to comply with giving oral sex during first-time sexual encounters (Katz & Schneider, 2015).

### The Present Research

Although several researchers suggest that sexual relations may be particularly fertile ground for the enactment of emotional labor (Elliott & Umberson, 2008), it has received surprisingly limited attention in sexuality research. In the present research, we aimed to develop and validate a multidimensional scale that captures the important aspects of emotional labor performed by women in their sexual lives. The five themes we reviewed above were central to the current study because they reflect the diverse ways in which women engage in emotional labor within their sexual experiences. All five themes identify behaviors that women enact in service of “faking” or adjusting their emotions to prioritize their partners’ needs and desires over their own. By providing this scale, we hope to offer a tool for investigating the role of emotional labor within partnered sexuality and a framework for considering gender inequality in our close relationships and intimate lives.

As part of the current research, we conducted three studies to develop and validate our scale. Study 1 aimed to create a scale to assess the multifaceted dimensions of sexual emotional labor and explore its factor structure. In Study 2, we sought to verify the identified factor solution determined in Study 1 and to explore first indicators of the scale’s reliability and validity. Finally, Study 3 aimed to validate the newly developed scale based on its relation to a broader range of variables, establishing comprehensive convergent and discriminant validity.

Given that emotional labor is primarily performed by women in predominantly heterosexual contexts (Fahs & Swank, 2016), our sample of Study 1 and Study 2 was limited to women in relationships with men. For Study 3, the sample consisted of heterosexual women who were either single or in relationships.

Data file, syntax, and study protocols of all studies are available on the OSF: https://osf.io/sqn9k/. The studies received IRB approval from the first author’s home institution. Study 2 and Study 3 were pre-registered^1^: Study 2: https://aspredicted.org/qdrh-4vsb.pdf, and Study 3: https://aspredicted.org/wrf5-82fm.pdf.

## Study 1

The primary objective of Study 1 was to establish an initial pool of items for a new measure of sexual emotional labor and reduce the preliminary item pool to a final list using exploratory factor analysis (EFA). Additionally, we aimed to assess the reliability of the newly developed scale. We expected the EFA to result in delineating five subscales capturing different aspects of sexual emotional labor: (1) faking orgasm, (2) tolerating discomfort or pain, (3) accepting dissatisfying or bad sex, (4) basing sexual satisfaction on partner’s pleasure, and (5) performing desire.

## Method

### Participants and Procedure

To select the sample size for our initial study, we applied the guidelines by Sakaluk and Short (2017) that consider 200–250 participants an optimal sample for exploratory factor analyses with moderately optimal data conditions. We recruited 257 women from the UK and the USA who are partnered with men via *Prolific*, an online research platform that enables researchers to reach their target participants due to thorough prescreening mechanisms. Participants were compensated with £0.85. We excluded eight participants who did not meet our inclusion criteria (i.e., cis-gender women, having a male partner).

The final sample consisted of 249 women (*M*_age_ = 40.31 years, SD = 12.23, range 19–80). Most women identified as heterosexual (92%), while the rest identified as bisexual. Participants’ relationship length ranged from 0.5 to 56.7 years (*M*_length_ = 16.42, SD = 11.33). Participants were instructed to answer all questions regarding their sexuality with their current partner. The dataset used in this study was complete, with no missing data. See Table 1 for a detailed sample description for all studies.

**Table 1:** Sociodemographic characteristics of participants across all studies

	Study 1		Study 2		Study 3	
	*N*	%	*N*	%	*N*	%
*Ethnicity*						
African American/Black British/Black	4	1.6	5	1.6	5	1.8
Asian American/Asian British/Asian	11	4.4	11	3.5	12	4.4
European American/Caucasian/White	228	91.6	276	89	241	88.6
Hispanic/Latina	1	0.4	4	1.3	4	1.5
Middle Eastern	1	0.4	4	1.3	3	1.1
Multi-ethnoracial	4	1.6	10	3.2	7	2.5
*Education*						
Less than high school	3	1.2	1	0.3	2	0.7
High school diploma	40	16.1	51	16.5	30	11
Some college	43	17.3	42	13.5	47	17.3
2-year college/university degree	32	12.9	35	11.3	30	11
4-year college/university degree	77	30.9	98	31.6	98	36
Master’s degree	35	14.1	54	17.4	45	16.5
Professional degree/Doctoral degree	19	7.6	29	9.4	20	7.4
*Political orientation*						
Extremely liberal	17	6.8	17	5.5	6	2.2
Liberal	82	32.9	83	26.8	70	25.7
Slightly liberal	30	12	48	15.5	44	16.2
Moderate	82	32.9	107	34.5	100	36.8
Slightly conservative	26	10.4	39	12.6	28	10.3
Conservative	11	4.4	12	3.9	21	7.7
Extremely conservative	1	0.4	4	1.3	2	1.1
*Country of residence*						
United Kingdom	240	96.4	306	98.7	268	98.5
USA	9	3.6	4	1.3	4	1.5

### Measures

#### Development of the Women’s Sexual Emotional Labor Assessment

Through an extensive review of the literature, we identified a few instruments that corresponded to specific aspects of our theoretical conceptualization of sexual emotional labor. For instance, we found scales addressing topics such as faking orgasms (e.g., Cooper et al., 2014). However, we were unable to locate a comprehensive measure that encompassed the multifaceted nature of sexual emotional labor. To fill this gap, we created an item pool covering different themes as suggested by the reviewed literature. Themes were largely derived from a selection of relevant qualitative research analyzing interviews with women (and men) about their experiences with emotion work in sexuality (Elliott & Umberson, 2008; Fahs & Swank, 2016; McClelland, 2010, 2014). See Table 2 for a list of all items.

**Table 2:** Factor loading and intercorrelations for both factor solutions

Item	First solution				Second solution			
	F1	F2	F3	F4	F1	F2	F3	F4
F1: Faking orgasm								
** I fake orgasms to protect my partner’s self-esteem/ego**	1.02				0.99			
** I fake orgasms to make my partner feel good**	0.98				0.94			
** I fake orgasms to protect my partner’s masculinity**	0.93				0.90			
** I fake orgasms to end sex**	0.81				0.79			
F2: Performing desire *(In order to please my partner…)*								
** I have sex even when I do not really feel like it**		.85				0.92		
** I try to make myself feel physical desire, even when I am not in the mood**		.77				0.74		
** I engage in sexual activities that I do not desire**		.70				0.66		
I exaggerate how good the sex is	.57	.31						
F3: Tolerating discomfort or pain *(When I experience discomfort or pain* *during sex…)*								
** I stop what we are doing (R)**			1.03				1.01	
** I continue having sex**			.67				0.68	
** I tell my partner about it (R)**			.60				0.61	
F4: Partner-referenced sexual satisfaction *(When we have sex…)*								
** My partner’s sexual satisfaction is more important than my own satisfaction**				1.01				0.98
** It is more important to me that my partner enjoys himself than that I enjoy myself**				.91				0.94
F5: Accepting dissatisfying or bad sex								
We have sex that does not feel especially pleasurable to me	.48							
I end sex when it is not pleasurable (R)			.40					

The items indicated in bold have been selected for inclusion in the final scale

*Theme 1: Faking orgasm.* As one of the key forms of emotion work during sexual behavior, we developed four items addressing various situational and partner-oriented motivations behind faking orgasm (e.g., “I fake orgasm to make my partner feel good”).

*Theme 2: Tolerating pain or discomfort*. The act of enduring or downplaying physical pain or discomfort during sexual activities was assessed using three items (e.g., “When I experience discomfort or pain during sex, I continue having sex”). By extending the item phrasing from pain to include discomfort, we aimed to ensure the applicability of the items to a wider range of women’s experiences.

*Theme 3: Accepting dissatisfying or bad sex.* Engaging in sexual encounters that lack pleasure and accepting them as normative was measured through two items (e.g., “I exaggerate how good the sex is”).

*Theme 4: Defining sexual satisfaction based on the partner’s pleasure.* The tendency to put the partners satisfaction above one’s own was measured with two items (e.g., “When we have sex, it is more important to me that my partner enjoys himself, than that I enjoy myself”).

*Theme 5: Performing desire.* Engaging in partnered sexuality even in the absence of desire was measured with four items (e.g., “In order to please my partner, I have sex even when I do not really feel like it”).

To help establish content validity, all newly developed items were evaluated by two established researchers from the USA who have published on the topic of women’s emotional labor in sexuality. The items were thoroughly revised by those experts, and their improvement suggestions were implemented for the final version. After assessing and synthesizing the evaluators’ feedback, we began an initial, iterative process of item elimination and reformulation to best capture the range of behaviors associated with engaging in sexual emotional labor. We decided on a set of 15 items (see Online Appendix A for a full list of original items). For two items (those measuring the fourth theme of basing one’s sexual satisfaction on partner’s pleasure), participants indicated their agreement on a 6-point scale from (1 = *strongly disagree*, 6 = *strongly agree*). For the remaining four themes, participants were asked how likely the behaviors were to occur in their sexual encounters with their partner (1 = *extremely unlikely*, 6 = *extremely likely*). The scale points denote the ends of the agreement and likelihood spectrum. We refrained from assigning labels to each individual scale point.

### Statistical Analysis

Drawing on the methodological suggestions provided by Sakaluk and Short (2017), which specifically address EFA practices in sex research, we applied maximum likelihood estimation with oblique rotation (promax), allowing correlations between the individual factors. Two statistical tests were calculated to determine data fit for EFA. First, Bartlett’s test of sphericity tested the null hypothesis that the correlation matrix equals an identity matrix. For this, we expected the null hypothesis to be rejected. Second, the Kaiser–Meyer–Olkin (KMO) measure of sampling adequacy tested the strength of partial correlations between the factors. KMO requires values of at least 0.8 for the data to be considered well fitted for EFA (Comrey & Lee, 1992; Tabachnick & Fidell, 2007). Further, skewness and kurtosis were checked for each item to test normality. Based on suggestions by Curran et al. (1996), items with a skewness of ≥ 2 and kurtosis of ≥ 7 were considered unsuitable for normality assumptions.

We also examined item communalities, which describes the proportion of variance in each item that is explained by the factors. Although some authors suggest that communalities should lie at least above 0.40 (Costello & Osborne, 2005), these suggested cut-off values for communalities vary greatly among different studies (Beavers et al., 2013; Eriksson & Humphreys, 2014). Consequently, those indicators were considered alongside others. For factor retention, we followed the suggestions by Sakaluk and Short (2017) combining several indicators like the eigenvalue-greater-than-one rule, the visual scree-test, and above all theoretical and interpretability arguments. In accordance with other studies and suggestions, items for the final version were selected based on two criteria: high factor loadings (greater than 0.5), and no cross loadings of above 0.3 (Brown, 2015; Schudson & van Anders, 2022; Tabachnick & Fidell, 2007). Subscale scores were derived by averaging the item scores corresponding to each factor. Additionally, the total score, reflecting the overall construct, was calculated by averaging the scores of all 12 items.

## Results

Our data were well fit for factor analysis as the Kaiser–Meyer–Olkin measure of sampling adequacy reached 0.83 and Bartlett’s test of sphericity was significant (*p* < .001). The data were also normally distributed, with none of the items showing values of ≥ 2 for the skewness or ≥ 7 for the kurtosis. The communalities for all but two of the items, both from the theme “Accepting dissatisfying or bad sex,” were greater than 0.4, which Costello and Osborne (2005) suggested as the minimal value, indicating that items were well explained by the underlying factors. We considered eliminating the two items with low communalities (“I end sex when it is not pleasurable,” “We have sex that does not feel especially pleasurable to me”), but as suggestions about cut-off values in communalities vary greatly (Beavers et al., 2013) we decided to include all 15 items in the first EFA.

The EFA extracted four factors with eigenvalues > 1 that accounted for 67.8% of variance. The Cattell’s scree-plot indicated a 5-factor solution. However, the fifth factor only explained 2.4% of additional variance and was theoretically not interpretable. Hence, according to suggestions of theoretical fit by Fabrigar et al. (1999), we considered the four-factor solution. The proposed theme of “Accepting dissatisfying or bad sex” could not be identified as a factor, as the two items belonging to this theme did not show loadings above 0.5 on any of the factors. Furthermore, one item developed for the theme of “Performing desire” loaded highly on two factors. Following our predetermined criteria, these three items were subsequently excluded from the analysis.

After excluding the three items, the final solution of the 12 remaining items explained 74.8% of variance. The Kaiser–Meyer–Olkin measure of sampling adequacy was 0.79, and Bartlett’s test of sphericity was statistically significant, *χ*^2^(66) = 2295.44, *p* < .001, indicating that the data were appropriate for factor analysis. The first factor (eigenvalue = 4.48, *M* = 2.17, SD = 1.41, *α* = 0.95) explained 37.3% of variance and was labeled “Faking orgasm.” The second factor (eigenvalue = 1.79,* M* = 2.80, SD = 1.30, *α* = 0.82) explained 14.9% of variance and included three items around the theme of “Performing desire.” The third factor comprised three items, explained 12.7% of variance (eigenvalue = 1.52, *M* = 2.25, SD = 1.11, *α* = 0.81) and was labeled “Tolerating discomfort or pain.” Lastly, the fourth factor (eigenvalue = 1.18, *M* = 3.30, SD = 1.51, *α* = 0.96) explained 9.8% of additional variance and included two items with the subscale labeled “Partner-referenced sexual satisfaction.”

The four WOSELA subscales correlated moderately with each other (*r*_range_ = 0.21–0.43, *p* < .001), whereas the WOSELA subscales correlated highly with the WOSELA total score (*r*_range_ = 0.55–0.80, *p* < .001). This suggests that the subscales were measuring different dimension of a broader underlying construct (Jozkowski & Peterson, 2014; Levant et al., 2012). Cronbach’s alpha for the 12-item total scale was 0.87. The intercorrelations of the four subscales and the total score are presented in Table 3.

**Table 3:** Correlations among subscales and total score for Study 1

	F1	F2	F3	F4
F1: Faking orgasm	–			
F2: Performing desire	.43**	–		
F3: Tolerating discomfort or pain	.21**	.24**	–	
F4: Partner-referenced sexual satisfaction	.31**	.40**	.23**	–
WOSELA Total score	.80**	.75**	.55**	.64**

**p < .01

Inter-item correlations ranged from 0.81 to 0.93 for Factor 1, from 0.62 to 0.73 for Factor 2, from 0.58 to 0.77 for Factor 3, and were at 0.91 for Factor 4. For the full-scale, inter-item correlations ranged from 0.31 to 0.71. Both the initial and the second factor solution can be found in Table 2.

Age was correlated with the total score, *r*(247) = − 0.15, *p* = .017. However, at the subscale level only “Faking orgasm” showed a significant correlation, *r*(247) = − 0.16, *p* = .014. There were no significant correlations with the WOSELA or its subscales with relationship length or political orientation.

## Discussion

Study 1 identified a four-factor solution for our sexual emotional labor scale and provided first indicators of good internal reliability for the subscales as well as the total scale. While the subscales offer insights into diverse expressions of sexual emotional labor, the total score, estimating the shared variance of these factors, offers an analysis of a more broad brush and comprehensive pattern. Notably, four of the five proposed themes that guided item generation were clearly reflected in the factor solution. However, the items related to the theme “Accepting dissatisfying or bad sex” did not form an independent factor nor did they exhibit above 0.5 loadings on any of the other factors. We ascribe the poor performance of the two items written for the factor “Accepting dissatisfying or bad sex” to the comprehensiveness of the factor. The concept of “narrating bad sex as acceptable” is a cognitive process developed over time, based on patterns encountered through personal experiences and through comparing these experiences with cultural norms, dominant discourses, and with the experiences of others in our social worlds (McLean, 2021; McLean & Syed, 2016; Schwab, 2020; Singer, 2004). Girls and women are inundated with messages about the quality of sex they can expect from different partners or in different contexts, and these messages are likely so insidious that they avoid conscious articulation (Conley & Klein, 2022). Devising a measure for such a latent construct requires deeper theorizing and more extensive scale development than the scope of the current paper. Future research should attend to longer-term processes, such as internalizing cultural norms or developing self-narratives, that contribute to understanding women’s “acceptance of bad sex.”

## Study 2

The primary objective of Study 2 (pre-registered) was twofold: First, to replicate the factor structure observed in Study 1 by applying confirmatory factor analyses (CFA), and second, to evaluate the reliability of the scale. In Study 2, we also examined initial indications of convergent validity. Due to brevity reasons and the replication of all correlations in Study 3, the results are available on the OSF.

## Method

### Participants and Procedure

We recruited 324 participants from *Prolific (*£ 1.6 compensation). Since 14 participants did not meet the inclusion criteria (i.e., cis-gender women, having a male partner), they were excluded from analysis. The final sample included 310 women from the UK and the USA (*M*_age_ = 43.63 years, SD = 12.51, age range 23–76). The great majority of participants identified as heterosexual (94.8%), while the other women identified as bi- or pansexual. Participants relationship length ranged from 0.5 to 54.0 years (*M*_length_ = 18.52, SD = 11.97). There were no missing data. For a detailed sample description, see Table 1.

### Measures

#### Women’s Sexual Emotional Labor Assessment

The 12 items of the final factor solution from Study 1 comprised the new Sexual Emotional Labor Assessment Scale. In Study 2, the subscales demonstrated high internal consistency: “Faking orgasm” (*α* = 0.94), “Performing desire” (*α* = 0.84), “Tolerating discomfort or pain” (*α* = 0.79), and “Partner-referenced sexual satisfaction” (*α* = 0.94). Cronbach’s alpha for the total scale for Study 2 was *α* = 0.87.

### Statistical Analysis

The confirmatory factor analysis was conducted using *AMOS 23* (Finch et al., 2016), employing maximum likelihood estimation procedures. Following the recommendations of Hu and Bentler (1998), we considered several model fit indices to assess the fit of the proposed model, including the root-mean-square error of approximation (RMSEA), the comparative fit index (CFI), and the standardized root-mean square residual (SRMR). We gave a preference to these indices over the *χ*^2^ goodness-of-fit test, as *χ*^2^ tests are heavily dependent on sample size and correlations among variables (e.g., Hooper et al., 2008). Fit can be considered acceptable if CFI is 0.90 or greater and RMSEA and SRMR values are 0.10 or lower (Hu & Bentler, 1998); and as excellent if CFI are 0.95 or greater and RMSEA and SRMR is 0.06 or less (Hu & Bentler, 1998; Kenny, 2020). In addition to the indices noted in our preregistration (RMSEA, CFI and SRMR), we also looked at the TLI fit indices to further corroborate our results. Mirroring the CFI, for the TLI we considered a value of 0.90 or higher as an indication of good fit (Kenny, 2020). We tested both the four-factor solution that was found in Study 1, as well as an alternative one-factor solution to compare model fit.

## Results

**Fig. 1:**
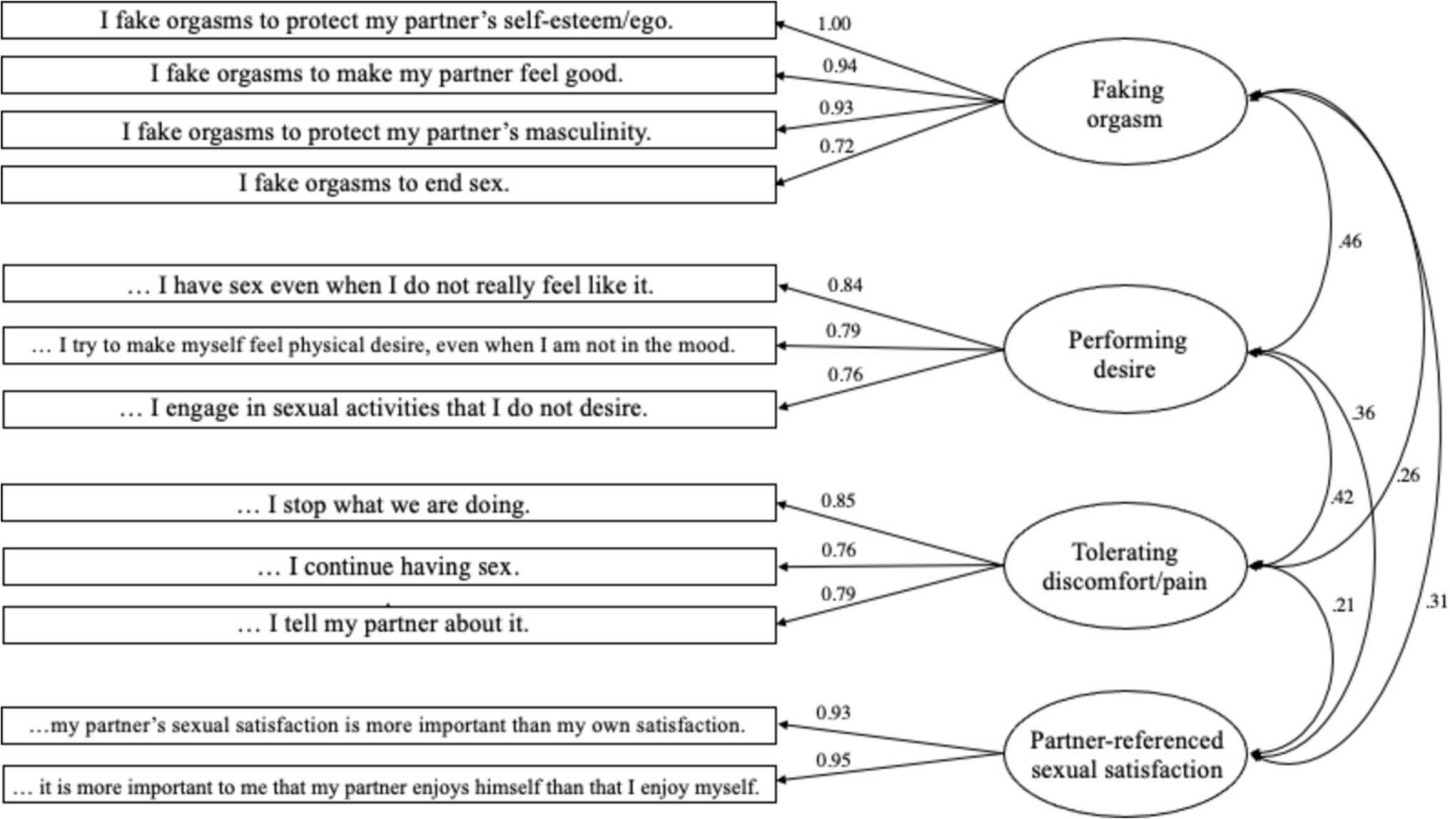
Standardized factor loadings and subscale correlations of confirmatory factor analysis

The results of the confirmatory factor analysis (CFA) provided evidence for the four-factor model identified in Study 1. The four-factor model provided an acceptable to excellent fit for the data, *χ*^2^(48) = 127.49, *p* < .001, RMSEA = 0.073 [0.058 − 0.089], CFI = 0.97, SRMR = 0.062, TLI = 0.96. Please see Fig. 1 for all standardized factor loadings of the four-factor model.

## Discussion

Study 2 replicated the four-factor structure of WOSELA and provided further evidence for the high internal reliability of the subscales, as well as the total scale.

## Study 3

In Study 3, we sought to further assess the factor structure’s stability, so we expanded the sample from women in relationships to include single women and conducted a CFA. We also incorporated various measures to examine the scale’s convergent and discriminant validity.

### Hypothesis 1 (H1)

We predicted that the WOSELA would be positively correlated with different constructs reflecting gendered scripts and norms. We expected a positive relationship between the WOSELA and (1) pleasure entitlement beliefs, (2) sexual script beliefs, and (3) feelings of duty to satisfy partner. Because emotional labor also involves the management of emotions and caregiving—which are traditionally understood as feminine values—we expected a positive correlation with (4) endorsement of feminine norms, providing evidence for the scale’s convergent validity.

### Hypothesis 2 (H2)

We further predicted the WOSELA to correlate with different measures of sexual behaviors, further demonstrating its convergent validity. Specifically, we expected a positive correlation with (1) submissive sexual behaviors, as well as negative correlations with (2) sexual agency, and (3) sexual communication, because engaging in emotional labor involves repressing one’s own thoughts and feelings, limiting sexual agency.

### Hypothesis 3 (H3)

We expected to find a negative relationship of the WOSELA and sexual and relational outcome variables, which would further confirm convergent validity. We predicted negative correlations of the WOSELA with (1) sexual pleasure experience, (2) sexual satisfaction, and (3) relationship satisfaction.

### Hypothesis 4 (H4)

Lastly, we hypothesized that the WOSELA will be unrelated to (1) sexual communal strength, and (2) partner focus in sexual desire, providing evidence for the scale’s discriminant validity.^2^

## Method

### Participants and Procedure

We collected data from 279 UK and US participants via Prolific* (*compensation £ 0.5). An a-priori power-analysis using G*power was conducted considering a small effect size for a bivariate correlation (*r* = 0.20) and revealed that approximately 266 participants would be needed to achieve 95% power. Seven participants did not meet our inclusion criteria resulting in a final sample of 272 women (*M*_age_ = 40.74 years, SD = 13.73, range 19–80). In Study 3, both self-identified heterosexual single women (15.8%) and women in relationships (84.2%) were included. Because gender stereotypes and gender roles permeate all facets of our society, we expected to find women performing emotional labor in sexuality across different relational contexts. There was no missing data.

### Measures

#### Women’s Sexual Emotional Labor Assessment

The WOSELA was applied as in Study 2, with the subscales “Faking orgasm” (*α* = 0.93), “Performing desire” (*α* = 0.84), “Tolerating discomfort or pain” (*α* = 0.78), and “Partner-referenced sexual satisfaction” (*α* = 0.94). Cronbach’s alpha for the total scale was 0.85.

#### Gendered Entitlement Beliefs

The belief that men are more entitled to sexual pleasure than women was measured with two items from previous research (Klein & Conley, 2022; Klein et al., 2024). Participants indicated their agreement with “Who has more of a right to experience orgasm during a sexual encounter?” and “Who needs an orgasm more during a sexual encounter?” Items were rated on a 10-point scale with two poles (1 = *woman*, 10 = *man*, *α* = 0.43). Given the low Cronbach’s alpha (*α* = 0.50), both items were again evaluated separately.

#### Sexual Script Beliefs

Gendered sexual scripts traditionally value men’s sexual pleasure over women’s sexual pleasure. We measured the extent to which participants endorse scripts supporting men’s entitlement to pleasure with the Sex-drive subscale of the Sexual Script Scale (Sakaluk et al., 2014, *α* = 0.93). The subscale includes five items (e.g., “Men need sex more than women”), which participants rate on a 6-point scale ranging from 1 = *strongly disagree* to 6 = *strongly agree*.

#### Feelings of Duty

We developed one item to measure feelings of sexual duty toward the partner: “It is a woman’s duty to satisfy her partner sexually.” The item was rated on a 6-point scale (1 = *strongly disagree*, 6 = *strongly agree*).

#### Feminine Gender Norms

Two items assessed participants internalization of gender norms (Wood et al., 1997). Participants were instructed to think about how society defines the ideal women. Afterward they were asked: “How important is it for you to be similar to the ideal woman?” and “To what extent is being similar to the ideal woman an important part of who you are?” Both items were measured on a 7-point scale (1 = *not at all*, 7 = *a great deal*). Cronbach’s alpha was 0.91.

#### Submissive Sexual Behavior

The preference for sexually submissive behavior was measured with four items (Sanchez et al., 2006; e.g., “I tend to take on the passive role during sexual activity”, *α* = 0.77). Participants indicated their agreement on a 7-point scale (1 = *strongly disagree*, 7 = *strongly agree*).

#### Sexual Agency

The Sexual Self-Efficacy in Achieving Desire and Pleasure subscale of the Female Sexual Subjectivity Inventory (Horne & Zimmer-Gembeck, 2006) was used to measure participants’ sexual agency. The scale includes three items (e.g., “I am able to ask my partner to provide the sexual stimulation I need”). Participants indicated their agreement on a 5-point scale (1 = *strongly disagree*, 5 = *strongly agree,* *α* = 0.91). We included an additional measure of sexual autonomy and agency that captures a broader sense of freedom and control with three items (Sanchez et al., 2005; e.g., “When I am having sex or engaging in sexual activities with someone, I feel free to be who I am”, *α* = 0.76). The items were measured on a 7-point scale (1 = *not at all true*, 7 = *very true*).

#### Sexual Communication

To measure women’s ability to communicate in the sexual context, we included the “Frequency” subscale of the Female Partner’s Communication during Sexual Activity Scale (McIntyre-Smith & Fisher, 2011). An example item is: “When having sex with a partner, how often do you ask your partner to stimulate your clitoris to orgasm?” Items were rated on a 6-point Likert scale (1 = *0% of the time*, 6 = *100% of the time*). Cronbach’s alpha for the scale was 0.80.

#### Sexual Pleasure

Sexual pleasure was measured with the three-item Sexual Pleasure Scale (Pascoal et al., 2016): e.g., “I find sexual intercourse…” All items are rated on a 7-point scale ranging from 1 = *not pleasurable* to 7 = *very pleasurable*. Cronbach’s *α* was 0.94.

#### Sexual Satisfaction

The overall satisfaction with one’s sexual life was measured with one item: “All things considered, how satisfied are you with your sexual life?” (Fischer & Traen, 2022). Participants rated their satisfaction on a 5-point scale ranging from 1 = *very dissatisfied*, 5 = *very satisfied*). A critical review of sexual satisfaction measures indicated that the single item showed good psychometric properties and can be considered a reliable and economic measure for sexual satisfaction (Mark et al., 2014).

#### Relationship Satisfaction

Participants’ satisfaction with their current romantic relationships was measured with a single item: “In general, how satisfied are you with your current relationship with your partner?” adapted from the Relationship Assessment Scale (Hendrick, 1988), on a 7-point scale (1 = *very dissatisfied*, 7 = *very satisfied*). We employed this single-item scale because thorough reviews have established the reliability and validity of this single-item measure of relationship satisfaction, as evidenced by its high correlations (ranging from 0.84 to 0.86) with the longer Relationship Assessment Scale (Fülöp et al., 2020).

#### Sexual Communal Strength

Sexual communal strength, which describes the motivation and willingness to meet a partner’s sexual needs and desires, was measured with the Sexual Communal Strength Scale (Muise et al., 2013). The scale consists of six items (e.g., “How far would you be willing to go to meet your partner’s sexual needs?”, *α* = 0.68) and was measured on a 5-point scale ranging from 1 = *not at all* to 5 = *extremely*.

#### Partner Focus in Sexual Desire

To measure the extent to which individuals’ sexual desire is motivated by fulfilling the needs of their partner, we applied the three-item subscale “Partner Focus” of the Sexual Desire Questionnaire (e.g., “When you experience sexual desire for a partner, is it generally characterized by a desire to make your partner feel happy?”; Chadwick et al., 2017). The items were rated on a 7-point Scale (1 = *strongly disagree*, 7 = *strongly agree,* *α* = 0.81).

## Results

### Confirmatory Factor Analysis

Again, the CFA provided good evidence for our four-factor model. The four-factor solution fit the data well, *χ*^2^(48) = 138.34, *p* < .001, RMSEA = 0.083 [0.067 − 0.100], CFI = 0.96, SRMR = 0.066, TLI = 0.95.

### Hypotheses Testing

Regarding H1, the WOSELA total score correlated positively but weakly with measures of gender script and norm beliefs. “Partner-referenced sexual satisfaction” showed weak but significant relations with the sex related gender norm variables, but not with general feminine gender norm endorsement. The latter was weakly correlated with “Faking orgasm” and “Performing desire,” while “Tolerating pain” was not significantly related to any of the variables (see Table 4).

**Table 4:** Descriptive statistics and correlations between the Women’s Sexual Emotional Labor Assessment (WOSELA) and gendered script and norm variables

	*M* (SD)	2	3	4	5	6	7	8	9	10
WOSELA										
1. Faking orgasm	2.30 (1.46)	.27**	.16*	.30**	.75**	.06	− .00	.04	.02	.13*
2. Performing desire	2.92 (1.34)	–	.37**	.33**	.71**	.11	− .02	.33**	.12	.13*
3. Tolerating pain or discomfort	2.16 (1.12)		–	.24**	.59**	.07	− .04	.07	.11	.10
4. Partner-referenced sexual satisfaction	3.32 (1.52)			–	.63**	.33**	.19*	.17**	.29**	.09
5. WOSELA_total_	2.60 (0.93)				–	.18**	.03	.21**	.16**	.17**
Scales for convergent validity										
6. Belief that men have more need for pleasure	5.93 (1.79)					–	.39**	.23**	.08	− .08
7. Belief that men have more right for pleasure	5.33 (0.98)						–	.15*	.13*	− .10
8. Sexual script beliefs	3.87 (1.31)							–	.14*	.08
9. Feelings of duty	2.54 (1.36)								–	.27**
10. Feminine norms	3.03 (1.64)									–

*p < .05, **p < .01

In support of H2, the WOSELA total score, the “Faking orgasm” subscale, and the “Performing desire” subscale showed positive correlations with sexual submissive behavior. Further, the WOSELA total score and all of the subscales were moderately to strongly negatively related to sexual agency measures as well as to sexual communication.

Consistent with H3, the WOSELA, as well as all of the subscales were negatively correlated with sexual pleasure experience. Additionally, three of the subscales were significantly negatively related to sexual and relationship satisfaction, while the “Partner-referenced sexual satisfaction” subscale showed no associations. Please see Table 5 for all correlations regarding H2 and H3.

**Table 5:** Descriptive statistics and correlations between the Women’s Sexual Emotional Labor Assessment (WOSELA) and sexual behavior and outcome variables

	*M* (SD)	6	7	8	9	10	11	12
WOSELA								
1. Faking orgasm	2.30 (1.46)	.12	− .17**	− .33**	− .13*	− .26**	− .21**	− .27**
2. Performing desire	2.92 (1.34)	.26**	− .22**	− .46**	− .22**	− .40**	− .22**	− .20**
3. Tolerating pain or discomfort	2.16 (1.12)	.18**	− .29**	− .40**	− .35**	− .28**	− .25**	− .20**
4. Partner-referenced sexual satisfaction	3.32 (1.52)	.11	− .21**	− .21**	− .27**	− .25**	− .05	.02
5. WOSELA_total_	2.60 (0.93)	.24**	− .31**	− .52**	− .33**	− .40**	− .28**	− .30**
Scales for convergent validity								
6. Submissive behaviors	4.29 (1.15)	–	− .21**	− .25**	− .32**	− .21**	− .07	− .18**
7. Sexual agency Horne and Zimmer-Gembeck (2006)	3.77 (1.01)		–	.47**	.66**	.51**	.33**	.26**
8. Sexual agency Sanchez et al., (2005)	5.44 (1.25)			–	.41**	.58**	.39**	.41**
9. Sexual communication	3.64 (1.30)				–	.49**	.34**	.29**
10. Sexual pleasure	5.50 (1.45)					–	.48**	.41**
11. Sexual satisfaction	3.57 (1.18)						–	.63**
12. Relationship satisfaction	6.03 (1.14)							–

*p < .05, **p < .01

Lastly, supporting H4, the WOSELA total score displayed no significant correlations with overall sexual communal strength and partner focus in sexual desire. Nevertheless, the subscale “Partner-referenced sexual satisfaction” demonstrated a weak correlation with both measures. On a closer examination at the item level of the Sexual Communal Strength Scale, our subscale exhibited significant correlation solely with one item: “How likely are you to sacrifice your own needs to meet the sexual needs of your partner?” (*r*(277) = 0.45, *p* = .021–< .001). Notably, it displayed no significant correlations with other items, all of which were partner oriented without the element of self-sacrifice. This item of the Sexual Communal Strength Scale was also significantly correlated to the other three subscales of the WOSELA (*r*(277) = 0.14–0.45, *p* = .021– < 0.001) (see Table 6).

**Table 6:** Descriptive statistics and correlations between the Women’s Sexual Emotional Labor Assessment (WOSELA) and sexual responsiveness variables

	*M* (SD)	6	7
WOSELA			
1. Faking orgasm	2.30 (1.46)	− .07	− .05
2. Performing desire	2.92 (1.34)	− .06	− .04
3. Tolerating pain or discomfort	2.16 (1.12)	− .02	− .07
4. Partner-referenced sexual satisfaction	3.32 (1.52)	.17**	.22**
5. WOSELA_total_	2.60 (0.93)	− .02	.00
Scales for discriminant validity			
6. Sexual communal strength	3.38 (0.58)	–	
7. Partner focus in sexual desire	5.48 (0.94)		–

**p < .01

There was no significant difference between single women (*M* = 2.58, SD = 1.05) and women in relationships (*M* = 2.61, SD = 0.91) in the WOSELA total score, *t*(270) = 0.22, *p* = .826. On the subscale level, there was only a significant difference for “Partner-referenced sexual satisfaction”, *t*(270) = 2.34, *p* = .020, with single women being less likely to endorse this type of emotional labor (*M* = 2.83, SD = 1.69) compared to women in relationships (*M* = 3.41, SD = 1.47).

## Discussion

Study 3 again confirmed the 4-factor structure of the WOSELA. We found additional support for the convergent validity of this new measure, particularly evident through its associations with sexual behavior and pleasure/satisfaction variables. Because we did not find a significant relationship between the WOSELA and positive-partner orientation (i.e., sexual communal strength), Study 3 demonstrates that this scale measures a distinct partner-oriented psychological phenomenon. Lastly, the mixed findings regarding sexual emotional labor and gendered norms and beliefs suggest both combining and distinct characteristics at play. While the endorsement of stereotypical gender norms did seem to be related to the enactment of certain types of emotional labor, the weak correlations imply that subscribing to these beliefs is not essential for women to engage in emotional labor. This subtlety underscores the concealed nature of emotional labor, hinting at the intricate dynamics that influence its manifestations.

## General Discussion

Emotional labor may play a significant role in shaping women’s sexual experiences, impacting their pleasure, agency, and overall satisfaction (Fahs & Swank, 2021). The purpose of the present research was to develop and validate a measure of women’s emotional labor in the context of sexuality. Based on previous qualitative research (Elliott & Umberson, 2008; Fahs & Swank, 2016; McClelland, 2010, 2014), the newly developed scale encompassed various crucial themes of sexual emotional labor. In Study 1, an exploratory factor analysis indicated a four-factor structure of the WOSELA with the subscales “Faking orgasm,” “Performing desire,” “Tolerating discomfort or pain,” and “Partner-referenced sexual satisfaction.” Studies 2 and 3 provided results of a confirmatory factor analysis supporting the model fit of this structure. Moreover, Study 3 demonstrated favorable psychometric properties of the WOSELA.

### Emotional Labor in Women and Its Associations with Women’s Sexual Well-Being

Throughout history, women’s pleasure has been consistently undervalued within heterosexual interactions (Conley & Klein, 2022; Frederick et al., 2018; Laan et al., 2021), and societal expectations place a strong emphasis on women being nurturing and attentive to their partner’s emotional needs (Crawford & Popp, 2003; Levant et al., 2012). This expectation of providing pleasure and the continuous management and monitoring of their own and their partner’s desires and emotions may greatly influence women’s sense of sexual agency (Cacchioni, 2007; Sanchez et al., 2006; Seabrook et al., 2017). In the present study, the WOSELA total score, as well as all subscales, showed moderate-to-high negative correlations with two different measures of sexual agency. One central aspect of behaving in a sexually agentic manner involves effectively communicating one’s needs and desires to a partner (Kiefer & Sanchez, 2007; Klein et al., 2018; Zimmer-Gembeck, 2013). Consistent with our predictions, results further revealed a negative correlation between the WOSELA and its subscales and women’s sexual communication behaviors. Rounding off the picture, the “Faking orgasm” and “Performing desire” subscales were also positively correlated with sexual submissive behavior, which is a key aspect of low sexual agency (e.g., Sanchez et al., 2006).

These findings are important because prioritizing the feelings of others can come at the cost of women’s own sexual agency, leading to a disconnection from their own needs, wants, and desires. Not only is sexual agency one of the strongest influencers of sexual satisfaction (Galinsky & Sonnenstein, 2011; Kiefer & Sanchez, 2007; Sanchez et al., 2006), but women who have a rather low sense of sexual agency are more likely to comply in sexual activities that they do not want (Darden et al., 2019; Fahs & Swank, 2021), putting them at risk of sexual violence or abuse. Sexual emotional labor could therefore be a potential barrier to women’s sexual autonomy. It is important to note, however, that women likely engage in sexual emotional labor without explicitly identifying it as such. In other words, while women might be aware of their involvement in specific behaviors (e.g., faking orgasm, performing desire) they may not understand these behaviors as constituting emotional labor.

Further, consistent with our predictions, we found a negative association between the WOSELA and its subscales and sexual pleasure. This falls in line with our proposed idea that emotional labor contributes to the sexual pleasure gap that disadvantages women. We also found that three of the WOSELA subscales were negatively related to sexual and relationship satisfaction. However, the “Partner-referenced sexual satisfaction” subscale showed no significant relation to these outcome variables. One plausible explanation for this is that individuals actively prioritizing their partner’s sexual needs over their own may be more inclined to gauge their own satisfaction in relation to their partner’s fulfillment (McClelland, 2010, 2014). This focus on the partner may elucidate why this subscale is linked to lower pleasure for women themselves, yet it does not necessarily lead them to perceive their overall sex life and relationship more negatively.

The present results add to past studies evidencing negative correlations between specific forms of emotional labor and sexual satisfaction and pleasure. For example, continuing penile-vaginal intercourse despite feeling pain (WOSELA factor: “Tolerating discomfort of pain”) has been linked to sexual dissatisfaction (Elmerstig et al., 2013), and complying with unwanted sex (WOSELA factor: “Performing desire”) has been associated with negative emotions, lower satisfaction and distress (Katz & Schneider, 2015; Muise et al., 2013). This supports the notion that the hidden emotional labor undertaken by women can significantly impact their sexual and relational well-being.

### Emotional Labor and Internalized Gendered Norms

If emotional labor stems from internalized gendered roles, it is likely practiced more automatically than consciously. In other words, even women who articulate a rejection of stereotypical gender roles may still perform the feminine role of enacting emotional labor in sexual contexts. To this end, we observed either no significant correlations or only weak associations between the WOSELA and constructs reflecting gendered norms and beliefs (e.g., internalization of feminine gender norms, the belief in gendered sexual scripts). These results indicate that the burden of sexual emotional labor is not solely confined to women who adhere to traditional gender roles. Consequently, emotional labor in the sexual domain is likely to be found among women who conform to, as well as among those who resist, traditional gender roles in other life domains. This aligns with previous research showing that almost all women in their studies had experienced engaging in some form of emotional labor in their sexual lives (Elliott & Umberson, 2008; Fahs & Swank, 2016).

One plausible explanation for these findings is the lengthy and deeply rooted history of gender stereotypes based on social roles, such as the expectation of women being caring, submissive, and non-demanding (Eagly & Wood, 2012). This normative influence transcends domains, shaping not only workplaces and domestic spheres but also intimacy and close relationships (Duncombe & Marsden, 1993; Umberson et al., 2015). Research shows that sexual contexts are likely to be especially influenced by gender norms, rendering it more challenging to dismiss internalized gender roles. For example, in priming experiments, participants who were presented with sexual cues more quickly self-categorized as a woman or man, identified more strongly with their gender, displayed increased self-stereotyping, and engaged in more gender-role behaviors compared to those in the control condition (Hundhammer & Mussweiler, 2012). Once these gendered roles are salient, resistance to situational expectations becomes difficult. The importance of perceived social expectations often compels individuals to conform to the situation and fulfill the presumed desires or expectations of others (Cooper & Withey, 2009), especially in the case of gender nonconformist behavior (i.e., backlash effects, Rudman, 1998).

### Emotional Labor and Sexual Responsiveness

Divergent validity of the WOSELA was demonstrated by the absence of a significant relationship with other partner-oriented measures of sexual responsiveness, specifically sexual communal strength, and partner focus in sexual desire. Both of these measures reflect positive sexual responsiveness, characterized by a motivation to fulfill a partner’s sexual desires (Muise et al., 2023). However, people who are high in sexual communal strength engage in partner-oriented behaviors for positive, autonomous reasons such as pleasure and intimacy rather than out of a feeling of obligation (Shoikhedbrod et al., 2022).

Although both sexual emotional labor and sexual communal strength involve engaging in behaviors to fulfill a partner’s desires, they differ in a crucial aspect: sexual emotional labor involves neglecting or suppressing one’s own emotions and desires, while sexual communal strength does not. Accordingly, our findings revealed that only one item from the Sexual Communal Strength Scale correlated significantly with all four subscales of the WOSELA: “How likely are you to sacrifice your own needs to meet the sexual needs of your partner?” This particular item captures the negative essence of prioritizing a partner’s needs above one’s own. This form of “negative” sexual responsiveness, mirrored in sexual emotional labor, may lead women to disconnect from themselves during sex, resulting in lower desire and satisfaction (Muise et al., 2023). In line with this, sexual emotional labor was negatively correlated to sexual and relational outcome variables, while communal strength has been linked to positive sexual outcomes in past research, such as higher sexual and relationship satisfaction and increased sexual desire over time (Impett et al., 2019; Muise et al., 2013).

### Challenges and Interventions for Reducing Sexual Emotional Labor

One of the biggest issues when trying to overcome the performance of emotional labor is the fact that it is performed internally, often automatically, and consequently is largely invisible (Hochschild, 1983). Although some aspects of sexual emotional labor may manifest through observable behaviors, such as faking orgasms, the cognitive work of aligning one’s actual emotions with their partner’s or cultural expectations occurs within the individual. The indirect, implicit aspects of psychological phenomena, such as emotional labor, make it difficult to identify, articulate, and evaluate as a practice. It is therefore plausible that women may assume this labor without explicit consent, negotiation, or recognition of its presence (West & Zimmerman, 1987). Accordingly, many women perceive negative sexual experiences as natural and expected (Bay-Cheng & Bruns, 2016; Conley & Klein, 2022). Thus, a crucial step toward overcoming emotional labor in the realm of sexuality involves raising awareness about its existence and its negative consequences for women’s sexual well-being.

Male partners can actively support this goal by challenging traditional masculine ideals of sexual performance, such as engaging in sexual activities and encounters with their female partners that explicitly depart from sexual scripts that prioritize male pleasure (Fahs & Swank, 2016). If a man could reassure his partner that his pleasure and orgasm are not goals to be attained at any cost, and/or that his self-confidence does not depend on her orgasm, his female partner would likely feel decreased pressure to engage in emotional labor such as sacrificing her pleasure for his or faking an orgasm. A recent study of feminist men highlighted that adopting feminist perspectives on sexuality, such as valuing equality in sexual pleasure, was associated with a broader range of sexual expression in men’s sexual relationships without diminishing their sexual desire (Son et al., 2024). The study also underscored the importance of open communication, mutual respect, and shared responsibility between partners as key factors in achieving sexual and relational equality.

### Limitations and Future Directions

Although our scale was developed based on research focusing on women’s heterosexual experiences, engaging in emotional labor might be also particularly prevalent within sexual minority groups. In the current research, we have primarily drawn on social role theory to suggest why women are more prone to perform sexual emotional labor compared to men. Following this line of thought, the internalization of feminine norms may persist within women, regardless of their partner’s gender. A qualitative study looking at emotional labor in long-term lesbian and heterosexual relationships found that women, regardless of their partners’ gender, performed more emotional labor than men, hinting toward a stronger influence of gender compared to relational context (Umberson et al., 2015). Future research should validate the scale in sexual minority groups (i.e., lesbian women, trans women) since we exclusively focused on heterosexual and cis-gendered contexts, which limits our study results. Given this focus, one item of the scale is not applicable to lesbian women as it references partner’s masculinity. Extending the application of the scale to sexual minority women would promote an understanding of the interplay between gender, sexual orientation, power, and emotional labor.

Intimate justice theory is a useful perspective for such an approach (McClelland, 2010). Intimate justice establishes a connection between experiences of inequity in the sociopolitical sphere and how individuals conceptualize and assess the quality of their sexual and relational experiences (McClelland, 2010, 2014). Personal experiences with sexuality are therefore affected by people’s social locations, including gender, class, race and/or sexual orientation. Those social locations are, in turn, connected to privileges, expectations and disadvantages that are conferred to those identities (McClelland, 2010; Zea et al., 2003). For instance, research indicates that not just women, but also sexual minority men often consider their partner’s sexual satisfaction when reporting on their own (McClelland, 2010). From an intimate justice perspective, it is very likely that emotional labor in sexuality may be a manifestation of power imbalances in sexual relations, regardless of subject-partner configuration.

Another limitation of this paper and our developed scale lies in its exclusive focus on emotional labor performed by women. This decision was based on existing research demonstrating that women tend to undertake a disproportionate share of emotional labor across various domains of life, including within the realm of sexuality (Dean et al., 2022; Fahs & Swank, 2016). Some research, however, indicates that men may also perform types of sexual emotional labor, for example by attempting to reduce their sexual interest and desire (Elliott & Umberson, 2008), or by faking orgasms (Wongsomboon et al., 2023). While recognizing its significant impact on women’s sex lives, future research could further enhance our understanding by expanding analyses to explore the role of emotional labor among men for a comprehensive approach.

The selection of sexual emotional labor themes was based on their recurrence in past qualitative analyses. These themes are certainly not exhaustive. Other forms of emotional labor in sexual behavior may include performative bisexuality, where women sexually interact with other women for the purpose of seeking validation (Fahs & Swank, 2016) or contraceptive responsibility, a concept referred to in the literature as “fertility work” (Bertotti, 2013) or “sexual safety labor” (Dutcher & McClelland, 2019). Contraceptive work underscores the disproportionate burden of avoiding pregnancy placed on women in heterosexual partnerships. Research indicates that women perform far more labor in this regard, facing physical burdens, such as hormonal side-effect, as well as investing time and attention (Bertotti, 2013; Dutcher & McClelland, 2019; Fennell, 2011; Littlejohn, 2013). In another expression of sexual emotional labor, women are frequently influenced by their partners’ pleasure when selecting contraceptive methods, such as opting for hormonal contraceptives over condoms to enhance their partners’ enjoyment (Higgins & Hirsch, 2008). Similar to other themes of emotional labor, this unequal burden is largely normalized, which might contribute to its hidden nature (Kimport, 2018). A promising avenue for future research could expand our proposed scale to incorporate additional emotional labor themes or investigating the relationship between the WOSELA and contraceptive behaviors, thereby further exploring the multifaceted nature of sexual emotional labor.

Another possible improvement for the WOSELA in future research is to enhance the clarity on the motivations underlying emotional labor behaviors in the item wording, particularly within the ‘Tolerating discomfort or pain’ subscale. While the primary reason for women tolerating pain during sexual activity is often the prioritization their partner’s pleasure (Carter et al., 2019; Elmerstig et al., 2013), other reasons that do not align with emotional labor are conceivable. To refine the subscale, we recommend considering the inclusion of a phrase such as “in order to please my partner.” Additionally, in our scale we did not include a specific time frame, which may have led to some ambiguity in participants responses. Future iterations could benefit from incorporating a reference, for instance, “within the last year” to enhance precision.

The WOSELA holds significant potential for diverse applications in future research endeavors to better understand gender inequalities in the intimate domain. For instance, employing the scale offers a unique opportunity to delve into the role of emotional labor in perpetuating the well-documented heterosexual pleasure gap. Despite decades of research on this phenomenon and the identification of many contributing factors (e.g., cultural overvaluing of penetration, lack of women’s entitlement), the inequality largely persists (Döring & Mohseni, 2022). Emotional labor may constitute a previously overlooked gendered factor sustaining the pleasure gap. Future research could explore the relevance of emotional labor in women’s sexual pleasure experiences, both in comparison to and in conjunction with other relevant factors, like entitlement or agency.

Exploring emotional labor in sexuality as a manifestation of power imbalances in sexual relations would be another interesting task for future research. To better understand women’s well-being, investigating the interconnection between women’s emotional labor in the context of sexuality and the performance of emotion work in other domains, such as childcare or within the relationship itself might be a crucial first step (Fahs & Swank, 2016).

Existing research suggests that embracing a feminist identity, as opposed to merely holding feminist values, is more likely to empower individuals to assert sexual agency (Bay-Cheng & Zucker, 2007). Future studies could consequently investigate potential protective factors that mitigate women’s engagement in emotional labor. While our study revealed a limited association between sexual emotional labor and the endorsement of gendered scripts and norms, there is a need to explore additional factors that may act as safeguards. Understanding such protective factors could provide valuable insights into promoting healthier and more equitable dynamics within the context of intimate relationships.

### Conclusion

Sexual emotional labor may be one of the most overlooked forms of gendered labor (Elliott & Umberson, 2008) and is likely to play a substantial, yet to date mostly hidden role in the perpetuation of the gendered pleasure gap. It was thus the goal of this research to develop a scale measuring this phenomenon to articulate its existence and make it measurable for quantitative research. The present set of studies introduced the WOSELA, a concise, yet broad measure that captures the multifaceted dimensions of emotional labor performed by women in the realm of sexuality. The WOSELA showed strong psychometric properties in terms of its factor structure, reliability, and validity. We hope this instrument can serve as a valuable tool for future research investigating the complex role of sexual emotional labor, especially in impacting women’s pleasure experiences.

## Supplementary Information

Below is the link to the electronic supplementary material.

Supplementary file1 (DOC 25 KB)

## Data Availability

Data files, syntax and study protocols of all studies are available at: https://osf.io/sqn9k/

## Footnotes

1 A deviation from the initial preregistration is discussed here: https://osf.io/km82d?

2 Initially, in Study 2, “Partner focus in sexual desire” (PFSD) was considered as a measure of convergent validity, as it was believed to align with the partner-oriented aspects captured by the WOSELA. However, based on the results obtained, along with further theoretical considerations, it was anticipated that there would be either no or only low correlations between PFSD and the WOSELA. This shift in thinking arose due to the recognition that the PFSD construct emphasizes positive-approach oriented aspects of sexual responsiveness, while the WOSELA is distinctive in prioritizing the needs of the partner above one's own, encompassing elements of self-neglect and capturing a gendered dynamic. This quality, unique to the WOSELA, is absent in the construct of PFDS. Consequently, PFSD was reevaluated for its potential to provide discriminant validity instead. Similarly, the construct of "Sexual communal strength" was also explored for its relevance to discriminant validity, as it also reflects a positive and autonomous motivation to fulfill a partner's needs.
