# Supplementary material for: The Intimate and Sexual Costs of Emotional Labor: The Development of the Women’s Sexual Emotional Labor Assessment
Source: Arch Sex Behav. 2024 Dec 19;54(1):117–38. doi: 10.1007/s10508-024-03061-7 (PMC11782323; doi:10.1007/s10508-024-03061-7)
Supplement: Supplementary file 1 — Supplementary file1 (DOC 25 KB) [file 10508_2024_3061_MOESM1_ESM.doc]

**Appendix A**

**Original Survey Items**

Please indicate the extent to which each of the following behaviors are likely to occur in the sexual encounters with your current partner.

Subscale: *Faking orgasm*

1. I fake orgasms to protect my partner’s masculinity.
2. I fake orgasms to protect my partner’s self-esteem/ego.
3. I fake orgasms to end sex.
4. I fake orgasms to make my partner feel good.

Subscale: *Tolerating discomfort or pain*

1. When I experience discomfort or pain during sex, I stop what we are doing. (R)
2. When I experience discomfort or pain during sex, I continue having sex.
3. When I experience discomfort or pain during sex, I tell my partner about it. (R)

Subscale: *Accepting dissatisfying or bad sex*

1. We have sex that does not feel especially pleasurable to me.
2. I end sex when it is not pleasurable. (R)

Subscale: *Performing desire* (In order to please my partner…)

1. … I have sex even when I do not really feel like it.
2. … I engage in sexual activities that I do not desire.
3. … I try to make myself feel sexual desire, even when I am not in the mood.
4. … I exaggerate how good the sex is.

*Scale: 1= extremely unlikely, 6= extremely likely*

Subscale: *Using partner’s satisfaction as a reference point for own satisfaction*

1. When we have sex, it is more important to me that my partner enjoys himself than that I enjoy myself.
2. When we have sex, my partner’s sexual satisfaction is more important than my own satisfaction.

Scale: 1 = *strongly disagree*, 6 = *strongly agree*
